# Advanced Analysis of Clay Microporosity Using High-Resolution N_2_-Ar Adsorption Isotherms Coupled with the Derivative Isotherm Summation Method

**DOI:** 10.3390/molecules29246019

**Published:** 2024-12-20

**Authors:** Anwar El Azrak, Denys I. Grekov, Laurent Truche, Pascaline Pré

**Affiliations:** 1 IMT Atlantique, GEPEA, UMR CNRS 6144, F-44307 Nantes, France; 2 Univ. Grenoble Alpes, CNRS, IRD, IFSTTAR, ISTerre, 38000 Grenoble, France

**Keywords:** clay, cation exchange, DIS method, microporosity, *t-plot*

## Abstract

The textural properties of synthetic and natural clays in the sodium form and exchanged with tetramethylammonium cations (TMA^+^) were characterized using N_2_ and Ar physisorption isotherms at cryogenic temperatures. Specific surface areas and micro/mesoporous volumes were determined using the BET and the *t-plot* models. The *t-plot* analysis requires the use of reference isotherms measured at the same temperature on the surface of non-porous materials with an identical chemical composition. In order to better assess the effects of chemical heterogeneities in the clay particles, reference isotherms representative of silica surfaces were taken into account in the analysis of the *t-curve* and corrected to account for variations in curvature at the interface of the film adsorbed in the micropores. In addition, high-resolution Ar adsorption isotherms at 87 K were analyzed using the Derivative Isotherm Summation (DIS) method to quantify the energy contributions of adsorption sites and determine the fractions of basal and lateral surfaces of clay particles. The high-energy adsorption sites, identified in the low-pressure range, were attributed to intra-particle microporosity due to stacking defects and/or open inter-layer spaces. These sites were differentiated from those on the lateral and basal surfaces of the particles. A modification of the DIS method was proposed to measure these contributions and improve the fit with the experimental data. The results show that TMA^+^ cation exchange significantly increases the microporosity of clays compared to their sodic form, which can be attributed to the increased contribution of intra-particle adsorption sites due to interlayer expansion.

## 1. Introduction

**Figure 1:**
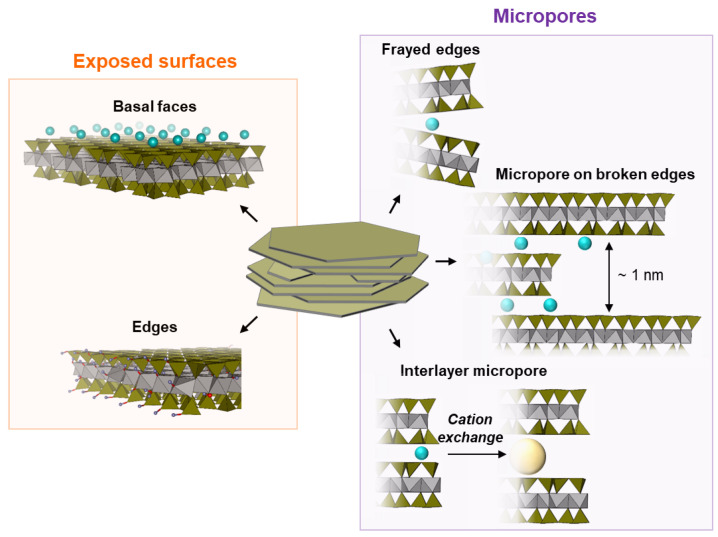
Key characteristics of clay texture at the particle scale.

The texture of clay minerals is determined by the stacking phyllosilicate sheets within the particles as a result of electrostatic interactions (e.g., smectites, vermiculites, micas) or hydrogen bonds (e.g., kaolinites) occurring between adjacent layers. The ordered stacking, characterized by a regular translation along the *c* axis and the alignment of the ditrigonal cavities of the tetrahedral layers, is often encountered in highly charged micas and muscovite. On the other hand, the turbostratic arrangement of the phyllosilicate sheets is typical of clays with a medium and especially low charge, such as smectites (Figure 1). The degree of sheet stacking disorder depends on the nature, location, and magnitude of the layer charge, on the type and hydration state of the charge-balancing cations, and sometimes also on the sample preparation conditions [1,2,3,4].

**Figure 2:**
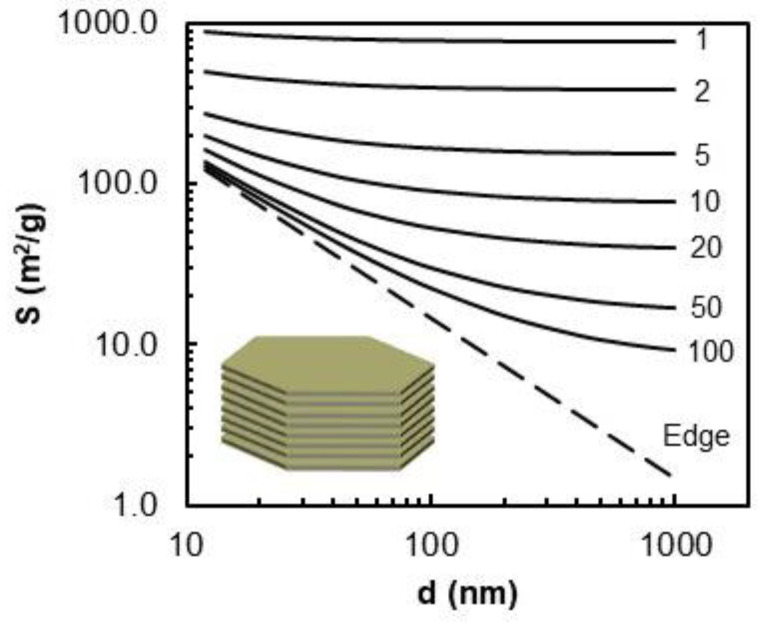
Theoretical surface areas of ordered clay crystallite as a function of dimensions of phyllosilicate sheets (*d*) and their stacking number (*n*) changing from 1 to 100.

The dimensions and arrangement of the phyllosilicate sheets in the clay particles define the primary texture of the clays, governing their surface area and microporosity. Taking into account the layering model, the theoretical outer surface area of the particle (geometric surface area) can be calculated as the sum of the basal and edge surfaces (1) [5].

(1) $$S_{particle}=S_{edge}+S_{basal}=\frac{4}{\rho_{s}\cdot d}\cdot 10^{6}+\frac{2}{\rho_{s}\cdot h\cdot n}\cdot 10^{6},\;\left[m^{2}\cdot g^{-1}\right]\;$$

where *ρ_s_* is the crystalline density, *d* is the diameter of the phyllosilicate sheet, *h* is the thickness of the phyllosilicate sheet (~0.95–0.97 nm, determined from *d_001_*), and *n* is the number of phyllosilicate sheets constituting the particle. The variation in the external surface area as a function of *n* and *d* for a smectite with *ρ_s_* = 2700 kg/m^3^ and *h* = 0.96 nm is illustrated in Figure 2. The surface of the edges is independent of the stacking and varies between ~100 and 1 m^2^/g, depending on the particles size.

The external surface area decreases considerably with the number of phyllosilicate sheets. The geometry of the clay particles can be described in terms of the surface fractions of the basal faces and edges relative to the total surface area or expressed in terms of the aspect ratio (2):

(2) $$Aspect\;Ratio\;\left(AR\right)=\frac{particle\;diameter\;}{particle\;height\;}=\frac{d}{h\cdot n}\;$$

Most natural clays have average particle sizes of less than 2 µm. Assuming a circular shape, a montmorillonite crystallite of 1 µm in diameter, comprising 10 stacked phyllosilicate sheets, has two basal faces (001) and a series of lateral faces (hk0) [6]. It also has a geometric surface area of about 80 m^2^/g, of which the edges represent 1.8%. In contrast, a 50 nm-diameter synthetic saponite or hectorite clay particle, composed of five stacked phyllosilicate sheets has a geometric surface area of ~200 m^2^/g with the contribution of edges of 20%. This assessment is approximate, since most of real systems have a turbostratic arrangement of phyllosilicate sheets and a distribution of the aspect ratio [7].

Intra-particle microporosity is associated with slit-shaped spaces on the broken edges or overlapping regions of phyllosilicate sheets stacked inside the particles [8]. Given the *d_001_* range for smectites, micropores in clays should have a nominal width of around 1 nm, with some degree of distribution due to the non-rigid nature of phyllosilicate sheets. The frayed edges and planar boundaries at the interface between two clay phases of interstratified minerals may also contribute to the formation of pores in the range of ultra-microprosity (<0.7 nm) [4]. For kaolinites [9,10] and several illite minerals [9], a comparison of the geometric surface areas measured directly via AFM with the specific surface areas derived from molecular probe physicorption data (BET model) showed that the results match well, with less than a 10% difference, which means that the specific surface area is mainly dominated by the outer faces of the clay particles. For smectites, on the other hand, 15 to 90% of the surface area can be located within the intra-particle micropores, so that their specific surface area is generally much higher than the geometric one [2,11].

In the dehydrated state, the intercalated spaces of the natural smectites are collapsed and inaccessible to the gas molecules [12]. One of the strategies for developing porous clay-based materials with a high surface area consists of pillaring the interlayer spacers with Al [13,14,15,16,17] polycations, bulky Cs^+^ ions [18,19], Ni^2+^ clusters [20,21,22], or protonated amines (1,4-diaminobutane—DAB, monomethylammonium—MMA, tetraethylammonium—TEA, tetramethylammonium—TMA) [23,24,25]. The latter group of pillaring agents makes possible the development of materials with sub-nanometric micropores, which can be interesting for gas treatment applications, such as CO_2_ capture or H_2_ storage.

The textural characterization of clays is essential for understanding the transport and sorption properties of natural clay systems, such as gas reservoirs in sedimentary basins [26], and deep underground nuclear waste deposits hosted in clay stones [27]. It is also crucial for the rational design of materials of interest for applications in catalytic or adsorption processes [12,28,29,30,31]. Probe molecule physisorption is widely used for the textural analysis of minerals and clay materials. Brunauer Emmet Teller (BET) and *t-plot* models applied to N_2_ adsorption isotherms at 77 K are commonly used to determine the specific surface area and micropore volume [1,5]. Derivative Isotherm Summation (DIS) is complementary to BET and *t-plot* analyses and is used to characterize the morphology of clay crystallites and their surface heterogeneities [32,33,34]. The DIS method is applied to determine the aspect ratio of the clay particles by modeling the high-resolution adsorption isotherms of the probe molecules. Because of their hydroxylated nature, the edges of the particles interact more strongly with probe molecules than the basal faces. As a result, the completion of the adsorbed films at the edges precedes the coverage of the basal planes, leading to the appearance of several components (inflections) on the high-resolution adsorption isotherms, which are highlighted by the derivation. The deconvolution of the simulated isothermal curve by a set of theoretical components contributing to the total specific surface area makes it possible to quantify these surface sites and is the origin of the name of the method—Derivative Isotherm Summation—DIS, introduced by Villieras and Michot for the characterization of clays and several other porous systems [32,33,34,35,36].

Based on a significant set of experimental data, three recommendations were made for the correct application of the DIS method to the textural characterization of clay systems [2,37]. First, N_2_ as a probe molecule is not suitable for assessing the clay aspect ratio due to its polarizability induced by the quadrupole moment. Adsorbed N_2_ molecules interact specifically with cations or hydroxyl groups on the surface of clay particles and exhibit orientation anisotropy due to their non-spherical shape. Second, for uncharged non-swelling clays, Ar adsorption gives accurate clay aspect ratios, as verified by cross-validation with AFM measurement data. Third, for charged non-swelling clays, the DIS method applied to high-resolution Ar isotherms returns correct basal and lateral surfaces, provided that surface-exchangeable cations are small monovalent species (Na, Li). Indeed, large and divalent cations can interfere with adsorbed molecules, affecting the evaluation of the various surfaces.

In the existing literature, the equilibrium [38] and quasi-equilibrium [32] adsorption isotherms of Ar at 77 K are exploited. However, under these conditions, Ar is 10 K below its boiling point and 7 K below its melting point, which makes it difficult to define its reference state and to establish appropriate assumptions defining the properties of the adsorbed phase [39]. 

In this work, the emphasis is on the methodological developments of the DIS method for the textural analysis of clays. High-resolution Ar adsorption isotherms measured at 87 K are used for the first time in the DIS analysis. They have been measured for a wide range of clay systems: non-swelling illite, a series of natural and synthetic smectites in Na^+^ and TMA^+^ exchanged forms [23]. The latter exhibited sub-nanometric micropores accessible to gas, which, to our knowledge, have never been characterized through DIS analysis. The intrinsic microporosity of different clay systems, independently evaluated using the *t-plot* method, was therefore taken into account in the deconvolution of the derivatives of the high-resolution Ar adsorption isotherms at 87 K in order to improve the quality of the fit with the experimental data.

## 2. Results and Discussion

### 2.1. N_2_ and Ar Adsorption Data

**Figure 3:**
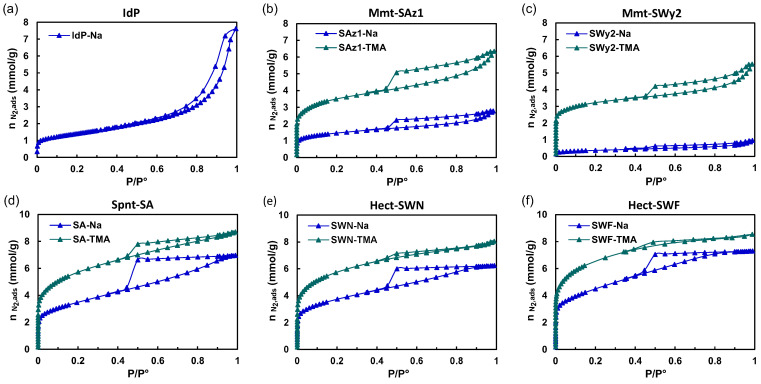
Nitrogen adsorption and desorption isotherms at 77 K for natural (upper) and synthetic (lower) clays exchanged with Na^+^ and TMA^+^.

Adsorption and desorption isotherms of N_2_ at 77 K for Illite du Puy (IdP), montmorillonites SAz-1, SWy-2, saponite SA, and hectorites SWF and SWN are shown in Figure 3.

The N_2_ isotherm of the sample IdP-Na (Figure 3a) is of type II(b) according to the classification given by [39], completed by Rouquerol et al. [40]. Such an isotherm is typical of clay structures composed of non-rigid aggregates of plate-like particles. The non-reversible adsorption branch associated with a narrow H3-type hysteresis loop indicates that monolayer-multilayer adsorption takes place on the outer surface of the particles up to a high P/P° (~0.8), followed by interparticle capillary condensation that induces cavitation during desorption in large pores (i.e., spontaneous nucleation and growth of gas bubbles in the metastable condensed fluid).

For montmorillonite clays, nitrogen isotherms are shown to be composites of type I and II(b) (Figure 3b,c), with shapes depending on the relative extent of the inter-lamellar and external areas [41]. We can therefore consider that the “gallery” pores (expanded interlayer spaces) of the forms exchanged with TMA^+^ contribute mainly to the range of micropores and are associated with pronounced nitrogen adsorption at a low P/P°. These isotherms also have an H4 hysteresis loop associated with capillary condensation in large mesopores. Since natural montmorillonites (Mmt) are swelling clays characterized by the disordered arrangement of crystallites composed of thin, flexible sheets, significant fractions of narrow-gallery micropores in the TMA^+^-exchanged forms should result from variable interlayer spaces able to locate adsorbed molecules. The H4 hysteresis loop observed on the N_2_ adsorption isotherms of Mmt SAz-1 (-Na, -TMA) and SWy-2 (-TMA) should be more representative of inter-particle mesoporosity. 

Regarding synthetic saponite and hectorite clays, nitrogen isotherms (Figure 3d–f) can also be described as composites of types I and II, corresponding to micro-mesoporous materials. However, the hysteresis loops of the isotherms representative of the Na-exchanged forms (SA, SWN, and SWF) seem to be of the H5 type, characterized by an ended step (saturation plateau), and suggesting the coexistence of open and partially blocked or constricted mesopores. Unlike natural montmorillonite samples, the exchange with TMA^+^ cations of these synthetic clays induces a significant reduction in the width of the hysteresis loop. This indicates less access to large pores, while increased adsorption in the low relative pressure range indicates a significant increase in the micropore volume. The apparent similarity of the shape and parallel alignment of isotherm regions associated with multilayer adsorption suggest that the structure of the secondary interparticle pores (i.e., large micropores and small mesopores) has undergone little change.

IdP has been extensively studied through DIS analysis, and a large amount of experimental data is available in the literature [9,38,42,43]. As shown based on cross-cheeked AFM and DIS measurements [9], IdP particles have 15–20% of the edges in their total surface area. This validated the reliability of the DIS for the quantitative assessment of the aspect ratio in this type of clay system and confirmed the assignment of the components of Ar isotherm derivatives.

**Figure 4:**
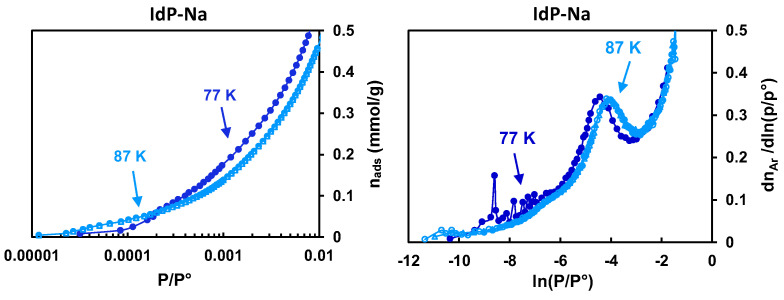
Ar adsorption isotherms (**left**) and their derivatives (**right**) at 77 K and 87 K for IdP-Na. The symbols in the form of circles and triangles correspond to two different series to evaluate the reproducibility of the results.

Figure 4 illustrates a comparison of Ar adsorption data for the IdP-Na at 77 and 87 K. The two curves are similar and have a major component and a secondary component in ln(P/P°) ranges −6 to −3 and −9 to −6, respectively, a shoulder below ln(P/P°) = −9, and an ascending profile above ln(P/P°) = −3. A slight shift between the maxima is induced by the effect of temperature. According to the conventional assignment, the main and the secondary components correspond, respectively, to the basal faces and to the edges, the component below ln(P/P°) = −9 is due to high energy sites on the edges, and the component greater than −3 is caused by a growth of multilayer adsorbed films on the external particles surface.

**Figure 5:**
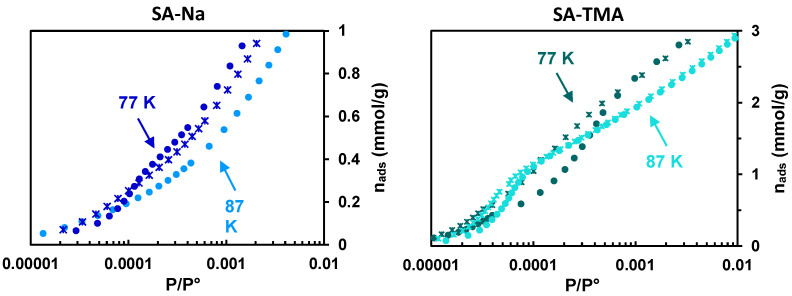

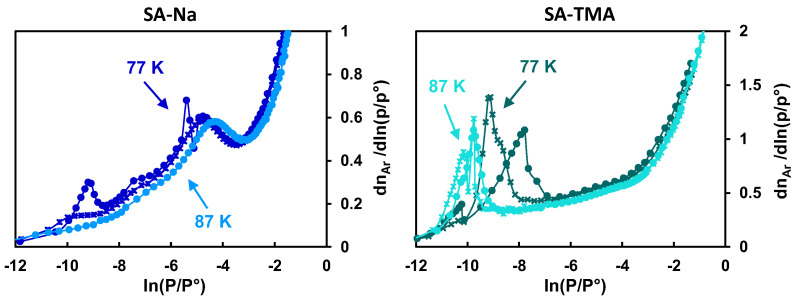
Raw Ar adsorption isotherms (**top**) and their derivatives (**bottom**) for SA-Na (**left**) and SA-TMA materials (**right**) at 77 K and 87 K. The symbols in the form of circles and asterisks correspond to two different series to evaluate the data reproducibility.

Argon adsorption isotherms measured for synthetic clays and several natural clays at 77 K, as illustrated for SA-Na in Figure 5 and for all the studied clays in Figure S1 (Supplementary Materials), show a non-reproducible inflection in the form of *s*-type loops at low pressure, which gives a sharp peak on the derivatives at ln(P/P°) < −7 with variable positions. This signature has not been documented earlier and can be attributed to an artifact. Indeed, at the initial stages of gas uptake, because the diffusion kinetics to adsorption sites are very slow, the equilibration time might not be reached, even after a 1 h duration of each acquisition point. To obtain reproducible isotherms, argon equilibrium data need to be collected at 87 K, corresponding to the gas probe liquefaction temperature at 1 bar. The repeatability of the experimental Ar adsorption isotherm data acquired in the low-pressure range could then be verified by tracing the adsorbed quantities and their derivatives as a function of ln(P/P°) for several runs (Figure 4 right). The resulting curves show no shoulder peak in the low-pressure range, and their shape has a profile similar to that of the reference system—IdP.

For the TMA^+^-exchanged clays (SA-TMA case illustrated in Figure 5), the repeated measurements of the Ar adsorption isotherms show relatively intense *s*-type loops (steps) in the low-pressure range, appearing more reproducible at 87 K than at 77 K. These loops lead to the appearance of peaks on the isotherm derivatives at ln(P°/P) < −8 and could be attributed to the contribution of the micropores formed by the widening of the interlayer spaces. By analogy with SA-Na, a better reproducibility of the derivative curves at 87 K can be explained by the achievement of the equilibrium conditions thanks to a faster diffusion kinetics of the Ar molecules in narrow micropores.

By comparing the derivatives of the Ar adsorption isotherm at 87 K for natural and synthetic clays, it can be seen that the major (−6 < ln(P/P°) < −3) and the secondary (−9 < ln(P/P°) < −6) components are obviously narrower for montmorillonite clays than for synthetic clays, probably because of their better crystallinity and less heterogeneous adsorption sites.

### 2.2. Characterization of BET Surface Area 

As shown in Table 1, cationic exchange of Na^+^ to TMA^+^ induces a significant increase in the specific surface area of all clay materials, from 170 to 230 m^2^/g. Such a result is relevant for more intense interactions due to the development of a novel gas-accessible microporosity that contributes either to monolayer or multilayer adsorption. Since the specific surface areas were determined with an estimated experimental error of ± 5%, the observed differences between the values obtained from Ar and N_2_ isotherms appear significant_._ The materials with the highest S_BET_ values are synthetic clay samples, for which the data derived from Ar isotherms measured at 87 K are lower than those obtained from N_2_ adsorption at 77 K. These differences can be explained by the anisotropy of the orientation of adsorbed N_2_ molecules and by the uncertainty in estimating the area occupied by a single molecule on the surface. In addition, due to their quadrupole moment, N_2_ molecules may exhibit specific interactions with surface functional groups or cations [39]. 

### 2.3. Characterization of t-Plot Micropore Volume 

In accordance with observed BET surface area variations, the microporous volumes deduced from the *t-plot* method, using either the conventional Harkins and Jura equation or the corrected silica mixed reference isotherms, are significantly larger for clays exchanged with TMA^+^ compared to the corresponding sodic counterparts. This phenomenon demonstrates the impact of cation exchange on the development of interlayer microporosity.

**Figure 6:**
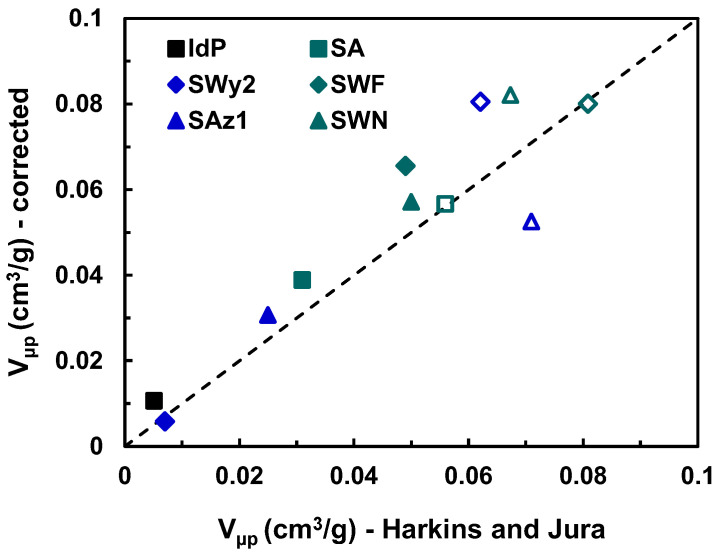
*t-plot* micropore volumes of the clay materials as determined from N_2_ physisorption data at 77 K using the Harkins–Jura equation [46] compared with the ones derived from the corrected reference thickness curve as proposed by Galarneau et al. [45]. Solid symbols represent clays in their sodium form, while empty symbols represent clays exchanged with TMA^+^.

Figure 6 shows the differences in microporous volumes between conventional and corrected reference isotherms used in the *t-plot* method. For some clays, whether they are slightly or highly microporous, there is a very good agreement between the two values. In contrast, four samples (SAz-1-TMA, SWN-Na, SWN-TMA, and SWF-Na) showed significant differences (greater than 0.02 cm^3^/g). Among natural clays, SWy-2 shows the greatest enlargement in its micropore volume after TMA^+^ exchange. Since the sodium form of SWy-2 has a micropore volume comparable to that of IdP-Na, such a variation can be explained by the swelling character of this clay, resulting in a larger cationic exchange capacity and a greater accessibility of the interlayer spaces [44]. However, other factors may influence the modulation of the clay micropore volume: congestion by exchanged cations, size, and degree of order of the layer arrangement. These results do not demonstrate that the correction of the *t-plot* model according to the reference curve equation provided by Galarneau et al. [45] improves the determination of the micropore volume of clay systems compared with the classical equation of Harkins and Jura.

For the well-crystallized natural montmorillonite systems, SWy-2 and SAz-1 exchanged with TMA^+^, the former shows a greater increase in the micropore volume compared to its Na form. At a constant interlayer opening, controlled by the TMA^+^ ionic diameter, a higher layer charge of SAz-1 (1.08 ē/unit cell vs. 0.6 ē/unit cell for SWy-2) [47] results in a higher concentration of TMA^+^ pillars in the interlayers and therefore in a reduction in the accessible space compared to SWy-2. Previous studies on these two clay systems have reported a similar trend [23]. In the case of semi-crystalline synthetic clays, featuring a lower layer charge than their natural counterparts, the development of the microporosity during TMA^+^ exchange is less significant, which could result from the rearrangement of the stacks, as often observed during the pillaring of similar systems [48] or from less accessible interlayer spaces. To answer this question, an in-depth structural characterization of the synthesized materials would be necessary.

### 2.4. DIS Characterization of Clay Surface Heterogeneity

The BET-H equation, proposed to describe the adsorption of probe molecules on exposed surfaces, is commonly used in DIS analysis. For the IdP-Na, the 87K Ar isotherm derivative can perfectly be fitted with a set of 4 BET-H components (cf. Section 3.5). As mentioned above, according to the commonly adopted assignment of DIS components for illite systems, the edges (component II) represent 18% of the total surface area, which is perfectly in accordance with the results reported in the literature [9,38,42,43].

**Figure 7:**
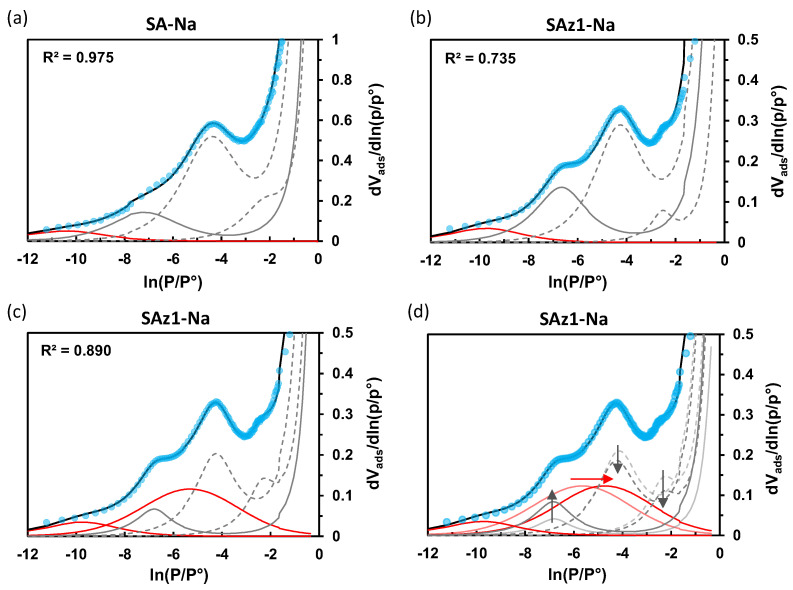
Derivatives of high-resolution Ar adsorption isotherms measured at 87K for SA-Na and SAz-1-Na (blue symbols) and best-fit simulations (black line) according to a conventional (**a**,**b**) and modified (**c**) DIS analysis. Individual components are assigned to adsorption on the basal (grey dashed line) and lateral (grey continuous line) faces of clay particles, and monolayer adsorption onto particle defective sites at the edges and within intra-particle microporosity (red lines). The effect of the position of the component related to intra-particle microporosity (red arrow) on the intensity of the components corresponding to the basal and lateral faces (grey arrows) is shown for the SAz-1-Na system in (**d**).

By analogy with IdP-Na, the experimental curves for the Na forms of the swelling clays presented in Figure 7a,b for SA and SAz-1 were initially simulated with a set of three BET-H and one BWT components. For the SA material, assuming that the peak at ln(P/P°) = −7 is due to edges, the fraction of the corresponding surface appears close to 20%, which is in a good agreement with the theoretical values depicted in Figure 2 for nano-clays. For SAz-1, however, the contribution of edges reaches 31%, which does not seem realistic, because according to the theoretical model, the surface of µm-sized particles in natural clays is mainly dominated by basal planes with a negligible contribution of edges (<5%). In addition, the isotherm fragment above ln(P/P°) = −3, corresponding to the multilayer adsorption, is greatly overestimated [49,50]. This means that the BET-H model is unable to fit properly the experimental data in this case. Similar discrepancies were observed for the other studied clays in their sodium form as reported in Figure S3.

As revealed by the *t-plot* method, the swelling smectite clays, especially in a TMA^+^ form, have a high microporosity, being accessible for Ar used as a probe molecule in the DIS analysis. As previously seen, the micropores in swelling smectite may include slit-shaped spaces located on the broken or frayed edges or in the regions that overlap on the stacked phyllosilicate sheets (Figure 1), in addition to interlayer spaces in the case of TMA pillared smectites. These surface domains should have higher adsorption energies than basal planes, so that their coverage with the adsorbed phase may precede the completion of the multilayer adsorption of Ar on exposed basal surfaces occurring above ln(P/P°) > −4. Thereby, the introduction of one or several BWT components suitable for the description of the microporosity resulting from such structural particle defects makes sense for the development of physically meaningful models of the clay texture. Under such an assumption, different components corresponding to the micropores should overlap in the low-pressure range. Figure 7c proposes an alternative fit for SAz-1-Na. The modified DIS model takes into account the micropore surface deduced from the *t-plot* data, in such a way that the total surface area of the components representing monolayer adsorption has been adjusted to be equal to the data deduced from the *t-plot* method using the Harkins and Jura reference curve (Table 2). The addition of this new BWT component improves the agreement with the experimental derivative curve in the high-pressure domain, as assessed by the determination coefficient R^2^. However, the position of this component is uncertain and affects the intensity of the peaks attributed to the contributions of the edges and basal planes, making the determination of the particle’s aspect ratio B/L indefinite (Figure 7d, Table 3).

**Figure 8:**
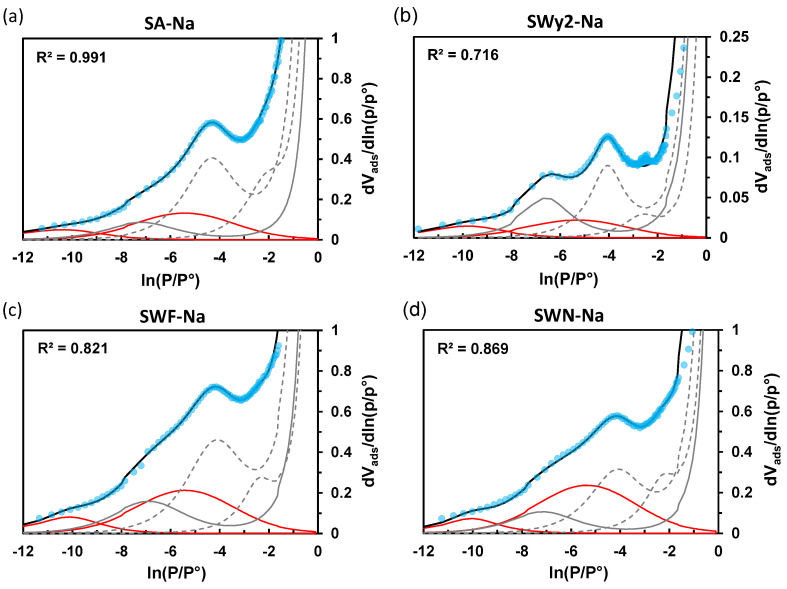
Derivatives of high-resolution Ar adsorption isotherms at 87 K (blue circles) for SA-Na (**a**), SWy2-Na (**b**), SWF-Na (**c**), and SWN-Na (**d**) together with their best-fit simulation with the modified DIS method (black line) and individual components attributed to adsorption on the basal (grey dashed line) and lateral (grey continuous line) faces of clay particles and monolayer adsorption onto defectives sites at the edges including micropores (red line).

Consequently, we have chosen to simulate all the derivative curves by fixing the position of this new component at an arbitrary value of ln(P/P°) = −5.5, intermediate between the range of low and high pressures. The results obtained for all clays in a sodium form using the modified DIS model are presented in Figure 8 and Table 4. This panel of clays includes systems with various crystallite sizes and B/L ratio values ranging from 1.27 to 5.34. A significant improvement is observed in the simulation of the experimental data in the high-pressure range compared to the conventional DIS method (Figure S3, Table S1, Supplementary Materials). The contribution of the multilayer adsorption on the edges then appears greater for clays with low B/L ratios. 

This procedure was also applied to clays exchanged with TMA^+^ in order to assess the contribution of the interlayer spaces to the microporosity (Figure S4, Table S2, Supplementary Materials). For SA, SWN, SAz-1, and SWy-2 shown in Figure S4a–c (Supplementary Materials), the large component in the lowest-pressure domain indicates the effect of cation exchange on promoting highly energetic adsorption sites in the expanded interlayers. For SWy-2 and SWF, the appearance of secondary components in the pressure range −10 < ln(P/P°) < −8, as shown in Figure S4d,e of the Supplementary Materials, could be due to complex diffusion mechanisms of adsorbate molecules in the interlayer spaces. The position and the amplitude of these secondary peaks may be associated with different energies of the corresponding adsorption sites located in the interlayer spaces.

Table S3 shows the surface areas of micropores (and defects on the edges) adsorbing Ar in the low-pressure range (at ln(P/P°) < −8). For clays exchanged with TMA^+^, these intra-particle microporosity areas are significantly increased compared to the corresponding Na-forms: by around 30% for natural clays and between 18% and 10% for synthetic clays.

## 3. Materials and Methods

### 3.1. Sample Preparation

Saponite SA, Hectorite SWN, and SWF in a Na-exchanged form were received from Kunimine Industries CO LTD (Tokyo, Japan). Natural montmorillonites SWy-2 and SAz-1 were purchased from The Clay Minerals Society (Chantilly, VA, USA).The chemical formulas of the studied clays are shown in Table 5. Natural montmorillonite samples have been purified to reduce the concentration of impurities, such as quartz, calcite, and other minor non-clay minerals. After dispersion in an acetic acid solution with a pH = 4.5 and stirring for 4 h to dissolve calcite, the clay slurry was subjected to 3 cycles of contact with a 0.2 M NaCl solution/centrifugation at 10,000 rpm for Na^+^ exchange, followed by 5 cycles of washing in deionized water (sequence of redispersion and centrifugation at 14,000 rpm) to remove excess salt. The concentration of clay was then adjusted to 15 g/L by adding deionized water, and the coarse fraction was separated via sedimentation and the slurry of fine particles was subsequently dried at 378 K, as recommended in the literature [51,52,53]. To exchange the sodium cations (ionic radius 1.02 Å) with tetramethylammonium ones (ionic radius 2.92 Å), all clays were brought into contact with a 0.2 M solution of the tetramethylammonium chloride (Fisher scientific TMACl (Fisher Scientific, Hampton, NH, USA): synthesis grade ≥ 98%), stirred for 12 h, and centrifuged at 10,000 rpm. The cycle was also repeated 3 times. The excess salts were removed by washing with large amounts of deionized water, and then, clays were suspended in a dialysis tube (SnakeSkin, 10 kDa MWCO, Fisher Scientific, Hampton, NH, USA) in deionized water for at least 7 days. Subsequently, clays were dried in an oven at 378 K and ground manually [18]. A Na form of Illite du Puy (IdP-Na) was used as non-swelling and non-microporous clay model [38].

### 3.2. Isotherm Measurements (N_2_, Ar)

Measurements of N_2_ and Ar (both gases purchased from Air Liquid (Paris, France) with a purity of 99.999%) adsorption–desorption isotherms were performed using a 3Flex automated static manometric adsorption analyzer from Micromeritics (Norcross, GA, USA), equipped with high-accuracy pressure transducers (precision 0.15% of absolute pressure reading) allowing for measurements (3Flex Version 5.00 software) in the domain of relative pressure ranging between 10^−7^ and 1. The acquisition was performed in a fixed-dose mode, varying the amount of injected gas between 0.01 and 10 cm^3^/g (STP), depending on the pressure interval and specific surface area of the analyzed sample. The equilibration time after gas injection was adjusted and optimized according to the protocol described in [38], so that high resolution isotherms could be collected, featuring at least 10–20 points per decade of gas pressure intervals. A dewar vessel filled with liquid nitrogen was used to maintain the temperature of the sample tube at 77 K during both N_2_ and Ar isotherm measurements. Ar isotherm data were furthermore collected above the triple point temperature, at 87 K, using a 3P Instruments cryoTune87 (Odelshausen, Germany) cryostat, so that the capillary condensation could occur. Prior to isotherm measurements, all samples, around 0.2 g each, were outgassed under a dynamic vacuum at 423 K until residual pressure was stabilized, for at least 12 h, as recommended in protocols described in [18,57]. The volumes of the cells not occupied by samples were determined after Helium expansion at room and cryogenic temperatures [51].

### 3.3. Specific Surface Area from the BET Model

Specific surface areas were determined from both N_2_-77K and Ar-87K adsorption isotherms using the BET theory [39]. The BET model was applied as specified by the IUPAC recommendations, assuming cross sectional areas of N_2_ and Ar equal to 0.162 and 0.138 nm^2^, respectively [58,59]. The selected pressure range for the BET surface area calculation was chosen in the domain of relative pressures 0.1 to 0.3 to fulfill the four consistency criteria of the BET equation, in accordance with Rouquerol et al. recommendations [40].

### 3.4. Micropore Volumes from the t-Plot Method

Fine characterization of micro-mesoporosity remains an open problem [60], although various methods have been developed [61]. The *t-plot* method is a widely used technique for determining the micro-mesoporous volumes and specific surface areas of porous materials. According to this method, the experimental isotherm data of the porous solid are used to plot the transformed *t*-curve describing the variations of the adsorbed amounts with the statistical thickness of the adsorbate film on the surface [62]. The relationship between the *t* thickness and the relative equilibrium pressure is deduced from the adsorption isotherm measured at the same temperature for a reference non-porous flat surface material having the same surface chemistry as the sample [45]. In the case of weak adsorbate/adsorbent interactions, the function *t* = f(P/P°) is independent of the nature of the solid surface. The Harkins–Jura Equation (3), which does not account for adsorbate/adsorbent interactions, is then convenient to be applied.

(3) $$t\left(\frac{P}{P_{0}}\right)\left(in\;nm\right)=0.1{\left(\frac{13.99}{0.034-log\left(\frac{P}{P_{0}}\right)}\right)}^{0.5}\;$$

In cases where surface interactions are specific, such as with zeolites, the reference curve must be representative of the surface chemistry heterogeneities. In the case of hierarchical micro-mesoporous zeolites, Galarneau et al. [45] have shown that to better describe variations in the film thickness in the low- and high-pressure domains, mixed reference isotherms should be determined from the adsorption isotherms of two silicas (microporous and mesoporous). After statistical analysis, the authors proposed to describe such mixed reference isotherms using the simple analytical functions expressed below (4):

(4) $$t\left(\frac{P}{P_{0}}\right)\left(in\;nm\right)=\left\{\begin{array}{c} A_{1}\left[1-exp(A_{2}P/P_{0})\right]+A_{3}\left[1-exp(A_{4}P/P_{0})\right],\;\;\;\;(P/P_{0}<0.03)\\ B_{1}{\left(P/P_{0}\right)}^{B_{2}}+B_{3}{\left(P/P_{0}\right)}^{B_{4}},\;\;\;\;\;\;\;\;\;\;\;\;\left(0.03\leq P/P_{0}<0.25\right)\\ {\left[\frac{C_{1}}{C_{2}-log(P/P_{0})}\right]}^{C_{3}},\;\;\;\;\;\;\;\;\;\;\;\;\;\;\;\;\;\;\;\;(0.25\;\leq \;P/P_{0}<0.6)\\ {\left[\frac{D_{1}}{D_{2}-log(P/P_{0})}\right]}^{D_{3}},\;\;\;\;\;\;\;\;\;\;\;\;\;\;\;\;\;\;(0.6\;\leq \;P/P_{0}<0.9)\\ E_{1}{\left(P/P_{0}\right)}^{E_{2}}+E_{3}{\left(P/P_{0}\right)}^{E_{4}}\;,\;\;\;\;\;\;\;\;\;\;\;\;\;\;\;\;(0.9\;\leq \;P/P_{0}) \end{array}\right.$$

where $A_{1}=0.1887299$ nm, $A_{2}=-481.3$, $A_{3}=0.182099$ nm, $A_{4}=-23.78$, $B_{1}=0.5675647$ nm, $B_{2}=0.199735$, $B_{3}=0.4116168$ nm, $B_{4}=2.00834$, $C_{1}=0.1423566$, $C_{2}=0.1078$, $C_{3}=0.4888$, $D_{1}=0.08309076$, $D_{2}=0.02995$, $D_{3}=0.369$, $E_{1}=1.268066$ nm, $E_{2}=1.931$, $E_{3}=0.76934$ nm, and $E_{4}=51.09$.

In addition, these authors, noting that the *t-plot* model underestimates the microporous volume of materials with a microporosity fraction greater than 20%, propose to correct the data deduced from the *t-plot* analysis using an abacus *y = f*(*x*), where $y=V_{µp}\left(t-plot\right)/V_{µp}Corr.$ and $x=V_{µp}/V_{total}\left(t-plot\right)\times 100$.

For *x* ranging between 20% and 60%, this corrective function can be expressed by the following Equation (5):

(5) $$y=A+Bx^{C}\;$$

where A=0.34, B=2.70, and C=−0.59.

The authors demonstrate that this correction takes into account the effect of curvature of the interface between the adsorbed film and the vapor phase, which cannot be neglected for small pore sizes (micro-mesopores with size < 10 σ, σ probe molecule size, σ = 0.36 nm for nitrogen and σ = 0.34 nm for argon). The curved interface results in an increase in the film thickness with a decrease in the pore size.

Since the clays studied are micro-mesoporous materials with polar surfaces likely to generate specific interactions, we chose to test this model to evaluate their porosity in addition to the conventional method using the Harkins and Jura equation.

### 3.5. Edge and Basal Specific Surface Areas from DIS

As introduced earlier, different surface domains of clay particles, such as particle basal and lateral faces, and micropores exhibit significantly different energies, so the adsorption of the molecular probe on these surfaces takes place in different domains of pressure, resulting in the appearance of several components on the isotherm. In case of overlap, multiple components can be better resolved using the derivation procedure. The DIS method consists in simulating the derivative of a high-resolution gas adsorption isotherm with the respect of ln(P/P°) using a set of theoretical isotherms able to assess the energetic characteristics and the contributions of the different surface domains to the total surface area. In particular, this technique is helpful for the evaluation of the aspect ratio, which is the ratio between the specific area of the edge and basal surfaces (B/L) of clay particles [33]. Ar physisorption is mainly employed for the characterization of clay minerals and clay materials using the DIS method. To progressively follow the formation of adsorbed films on different surface domains of clay particles, the DIS procedure requires smooth isotherm curves, collecting a high number of experimental points in the pressure range between 10^−1^ and 10^4^ Pa. Bragg–Williams–Temkin (BWT) and Brunauer Emmet Teller Hill (BET-H) (Equations (6) and (7) respectively) are commonly considered in the DIS analysis for high-resolution isotherm modeling. The BET-H model describes multilayer adsorption on exposed surfaces, simulating the coverage with probe molecules of the edges and basal planes (Figure 1). The BWT model, which is derived from the Langmuir assumptions, is representative of monolayer adsorption and is suitable to account for the contribution of the microporosity that may result from broken or frayed edges or particle interlayer spaces (Figure 1).

**Figure 9:**
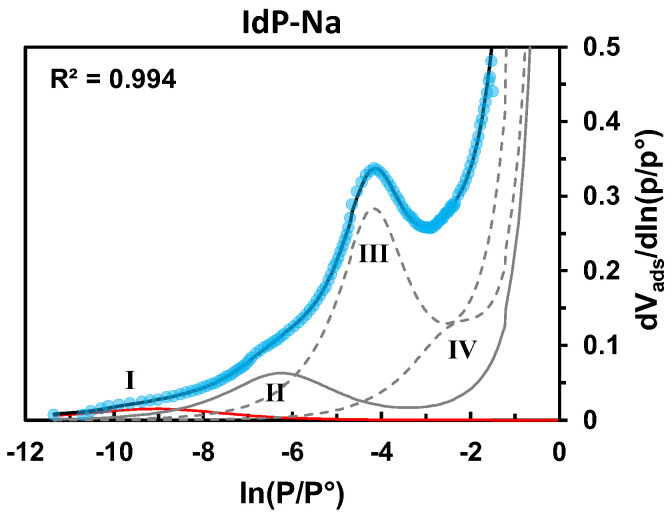
Derivative of high-resolution Ar adsorption isotherms at 87 K for IdP-Na (blue symbols) with its best-fit simulation (black line) and individual components attributed to adsorption on the basal (III, IV-grey dashed line) and lateral (II-grey continuous line) faces of clay particles and monolayer adsorption onto defective sites at the edges (I-red line).

Both of these equations contain terms *C* and $exp\left(\frac{\omega \cdot \theta }{\mathrm{kT}}\right)$, the adjustment of which changes the position and width of the simulated peaks of the isotherm derivative curve as illustrated in Figure S2, Supplementary Materials. These terms are relevant of adsorbate–adsorbent and lateral adsorbate–adsorbate interaction forces. According to the conventional assignment of the DIS signature of clays, as shown for well-crystallized illite and kaolinite clays characterized by a stacked plate particle geometry with closed interlayer spaces, the modeling of the low-pressure isotherm component, involves high values of *C* and low values of *ω/kT* because of strong adsorption interactions with high-energy surface sites on the edges of particles, also featuring a high structural disorder degree; this is exemplified for Illite de Puy-(IdP-Na) [38] in Figure 9 and Table 6.

In the low-pressure domain, the BWT equation (monolayer adsorption) simulates the isotherm derivate, while in the medium- and high-pressure domain, the BET-H equation depicts multilayer adsorption, which is supposed to take place over exposed particle basal planes and lateral faces (Figure 9) [38,63,64]. Both equations are solved using an iterative method for each experimentally measured pressure point. The derivatives of the experimentally measured isotherms are calculated using a numerical procedure [38]. The number of components needed to simulate the experimental isotherm derivatives, their weight, and parameters *C* and *ω* (Figure S2, Supplementary Materials) were adjusted as best-fit parameters minimizing the residual sum of squares between experimental and simulated curves, as recommended by Villiéras et al. [33]. The weights of the individual components correspond to their contribution to the total specific surface area of the porous material.

(6) $$\theta =\frac{C\cdot exp\left(\frac{\omega .\theta }{kT}\right)\cdot P/P^{{}^{\circ}}}{1+C\cdot exp\left(\frac{\omega .\theta }{kT}\right)\cdot P/P^{{}^{\circ}}}\;$$

(7) $$\theta =\frac{C\cdot exp\left(\frac{\omega \cdot \theta }{kT}\right)\cdot P/P^{{}^{\circ}}}{(1-P/P^{{}^{\circ}}).\cdot \left[1+(C\cdot exp\left(\frac{\omega .\theta }{kT}\right)-1)\cdot P/P^{{}^{\circ}}\right]}\;$$

where *θ* corresponds to surface coverage (ratio of adsorption capacity of molecular probe at given P and its monolayer capacity under the same temperature T), *C* is the energy constant corresponding to the adsorbate-adsorbent interaction, and *ω* is the parameter responsible for adsorbate–adsorbate interactions.

## 4. Conclusions

This work studies the effect of cation exchange on the evolution of textural properties of various natural and synthetic clays. Complementary modeling approaches were used, based on the measurement of nitrogen and argon isotherms at a cryogenic temperature. The first approach was to determine specific surface areas and microporous volumes, using the BET model and the *t-plot* method associated with the Harkins and Jura equation or the mixed equation proposed by Galarneau et al. [45]. Cation exchange with TMA^+^ induced a strong increase in the microporosity of clays relative to the corresponding Na-forms. The use of a reference curve, which takes into account the specific interactions created by the surface heterogeneities, induces a minimal change in the estimated values of the volumes and the surfaces of the micropores according to the *t-plot* equations proposed by Galarneau et al. [45]. The second approach consisted of the textural analysis carried out by modeling the high-resolution Ar isotherm derivatives measured at 87 K, using the DIS (Derivative Isotherm Summation) deconvolution method proposed by Villiéras [33]. It was shown that the conventional approach of DIS modeling (supposing multilayer adsorption on the lateral and basal surfaces) correctly describes the experimental data for sodium-exchanged clays, having a small fraction of the surface of micropores with respect to the total specific surface area. Conversely, for TMA^+^-exchanged clays, characterized by a higher microporosity, the conventional DIS method appeared insufficient to describe the contributions of high-energy adsorption sites in the low-pressure range. A modified approach was proposed to account for the additional contributions of adsorption sites located at structural edge defects (e.g., frayed or broken edges) and within the interlayer spaces of TMA-pillared clays. This was achieved by explicitly modeling these contributions as a monolayer adsorption component in the fitting process. The modified modeling approach offers an improved fit for describing the experimental isotherm derivative curves; however, the exact positioning of the deconvoluted peaks representing these contributions remains uncertain. Our results show that cation exchange with TMA^+^ significantly enhances the sorption capacity of clays, increasing intra-particle microporosity surfaces by up to 30% for natural clays and between 10% and 18% for synthetic clays, compared to their sodic form.

## Figures and Tables

**Table 1:** BET surface areas of the clays studied, measured from Ar isotherms at 87 K and N_2_ isotherms at 77 K.

Sample		S_BET_ (m^2^/g)—N_2_	S_BET_ (m^2^/g)—Ar
IdP-Na		112	100
SWy-2	Na^+^	30	44
	TMA^+^	262	178
SAz-1	Na^+^	117	120
	TMA^+^	288	249
SA	Na^+^	277	255
	TMA^+^	462	374
SWF	Na^+^	360	297
	TMA^+^	536	425
SWN	Na^+^	300	245
	TMA^+^	467	384

**Table 2:** Volumes of micropores calculated using the *t-plot* method applied to nitrogen isotherms measured at 77 K.

Sample		V_tot_ (cm^3^/g)	*t-Plot* (Harkins and Jura)		*t-Plot* (Corrected Ref. Isotherms [45])	
			V_µp_ (cm^3^/g)	S_µp_ (m^2^/g)	V_µp_ Corr. (cm^3^/g)	Corr. % _µp_
IdP-Na		0.243	0.005	11.53	0.011	4.4
SWy-2	Na^+^	0.027	0.007	15.64	0.006	21.6
	TMA^+^	0.173	0.062	147.94	0.08	46.6
SAz-1	Na^+^	0.087	0.025	59.58	0.031	35.4
	TMA^+^	0.201	0.071	172.30	0.053	26.1
SA	Na^+^	0.236	0.031	72.46	0.039	16.5
	TMA^+^	0.295	0.056	137.10	0.057	19.2
SWF	Na^+^	0.253	0.049	112.80	0.066	25.9
	TMA^+^	0.288	0.081	201.84	0.08	27.8
SWN	Na^+^	0.215	0.05	118.33	0.057	26.6
	TMA^+^	0.267	0.067	165.71	0.082	30.7

**Table 3:** Results of the DIS analysis applied to Ar adsorption isotherms at 87 K for SA-Na and SAz-1-Na: conventional and modified fitting approaches.

Component	Parameter	SA-Na	SAz-1-Na	Modified DIS Fit SAz-1-Na
I	*C*	27,000	12,000	12,000
	*ω/kT*	0	0.3	0.3
II	*C*	1000	440	360
	*ω/kT*	0.3	1.05	1.72
III	*C*	55	41	320
	*ω/kT*	0.75	1.1	−1
IV	*C*	5.6	4.3	33
	*ω/kT*	1.1	1.96	1.45
V	*C*	-	-	4.5
	*ω/kT*	-	-	1.55
Edge %		21.03	30.82	17.74
B/L		3.75	2.25	4.64

**Table 4:** Results of best-fit simulation with modified DIS method applied to Ar adsorption isotherms at 87 K for Na^+^ form clays.

Component	Parameter	Modified DIS			
		SWy2-Na	SA-Na	SWN-Na	SWF-Na
I	*C*	12,000	27,000	12,000	15,000
	*ω/kT*	0.3	0	1	0.7
II	*C*	320	1000	900	800
	*ω/kT*	1.28	0.3	0.4	0.2
III	*C*	320	320	320	320
	*ω/kT*	−1	−1	−1	−1
IV	*C*	23.2	45	38	43
	*ω/kT*	1.75	1	0.91	0.74
V	*C*	7	4.9	4.7	5
	*ω/kT*	1.4	1.13	1.38	1.5
Edge %		44.08	15.76	21.59	24.45
B/L		1.27	5.34	3.63	3.1

**Table 5:** Structural formula and cation exchange capacity (CEC) of clay precursors based on literature data.

Name	Structural Formula	CEC (meq/g)	Ref
SWy-2	(Na_0.16_Ca_0.06_K_0.025_)[Al_1.5_Fe(III)_0.21_Mg_0.27_Mn,Ti_0.02_][Si_3.99_ Al_0.01_]O_10_(OH)_2_	0.76	[47]
SAz-1	(Na_0.18_Ca_0.19_K_0.01_)[Al_1.36_Mg_0.55_Fe(III)_0.6_Mn,Ti_0.02_][Si_4.0_]O_10_(OH)_2_	1.2	[47]
IdP	(X(I)_0.12_)(Ca_0.01_Na_0.01_K_0.53_)[Al_1.27_Fe(III)_0.36_Mg_0.44_][Si_3.55_Al_0.45_]O_10_(OH)_2_	0.2	[54]
SWN	(Na_0.33_)[Mg_2.67_Li_0.33_][Si_4_]O_10_(OH)_2_	0.43	[55]
SWF	–	0.7	-
SA	(Na_0.25_Mg_0.07_)[Mg_3_Al_0.015_][Si_3.6_Al_0.4_]O_10_(OH)_10_	0.66	[56]

**Table 6:** Results of DIS applied to Ar adsorption isotherms at 87 K for IdP-Na.

Component	Parameter	IdP-Na	% Edges	B/L
I (defects on edges)	*C*	8000	18%	4.3
	*ω/kT*	0		
II (edges)	*C*	360		
	*ω/kT*	0.64		
III (basal)	*C*	30		
	*ω/kT*	1.50		
IV (basal)	*C*	7.20		
	*ω/kT*	1		

## Data Availability

The original contributions presented in the study are included in the article/Supplementary Material; further inquiries can be directed to the corresponding author/s.

## Supplementary Materials

The following supporting information can be downloaded at: https://www.mdpi.com/article/10.3390/molecules29246019/s1, Figure S1: Derivatives of Ar adsorption isotherms; Figure S2: DIS modeling: the effect of parameters of BWT model on isotherm derivative shape; Figures S3 and S4: Derivatives of high-resolution Ar adsorption isotherms at 87 K; Table S1: Comparison between results of conventional and modified DIS method; Table S2: Results of best-fit proposed of modified DIS method; Table S3: Intra-particle microporosity surfaces for the exchanged clays.
