# Supplementary material for: Advanced Analysis of Clay Microporosity Using High-Resolution N2-Ar Adsorption Isotherms Coupled with the Derivative Isotherm Summation Method
Source: Molecules. 2024 Dec 20;29(24):6019. doi: 10.3390/molecules29246019 (PMC11676051; doi:10.3390/molecules29246019)
Supplement: Supplementary file 1 [file molecules-29-06019-s001.zip › molecules-3352744-supplementary.pdf]

Advanced Analysis of Clay Microporosity Using High-Resolution N_2-Ar Adsorption Isotherms Coupled with the Derivative Isotherm Summation Method

Anwar El Azrak [1], Denys I. Grekov [1,*], Laurent Truche [2] and Pascaline Pré [1,*]

[1] IMT Atlantique, GEPEA, UMR CNRS 6144, F-44307 Nantes, France
[2] Univ. Grenoble Alpes, CNRS, IRD, IFSTTAR, ISTerre, 38000 Grenoble, France
* Correspondence: denys.grekov@imt-atlantique.fr (D.I.G.); pascaline.pre@imt-atlantique.fr (P.P.)

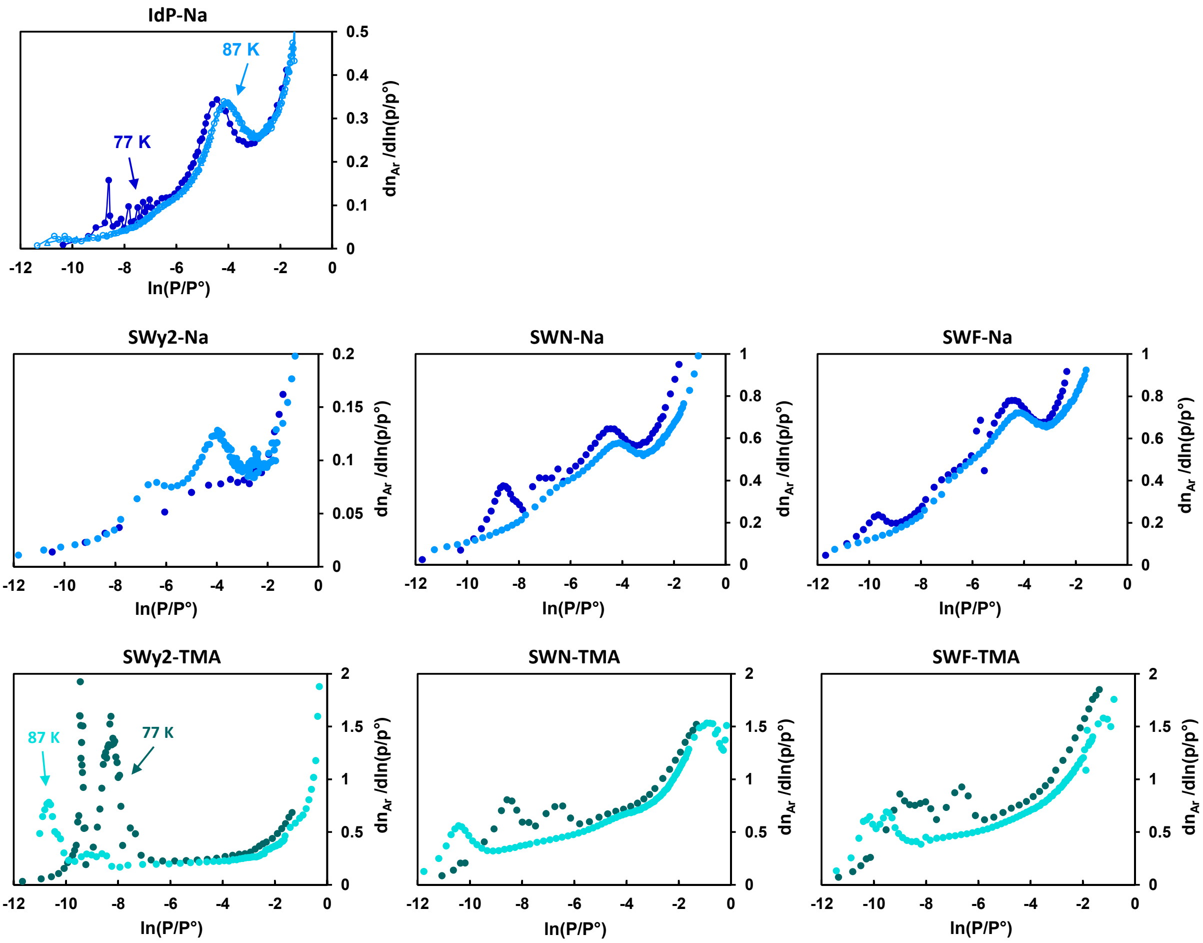

Figure S1. Derivatives of Ar adsorption isotherms for all studied materials at 77 and 87 K.

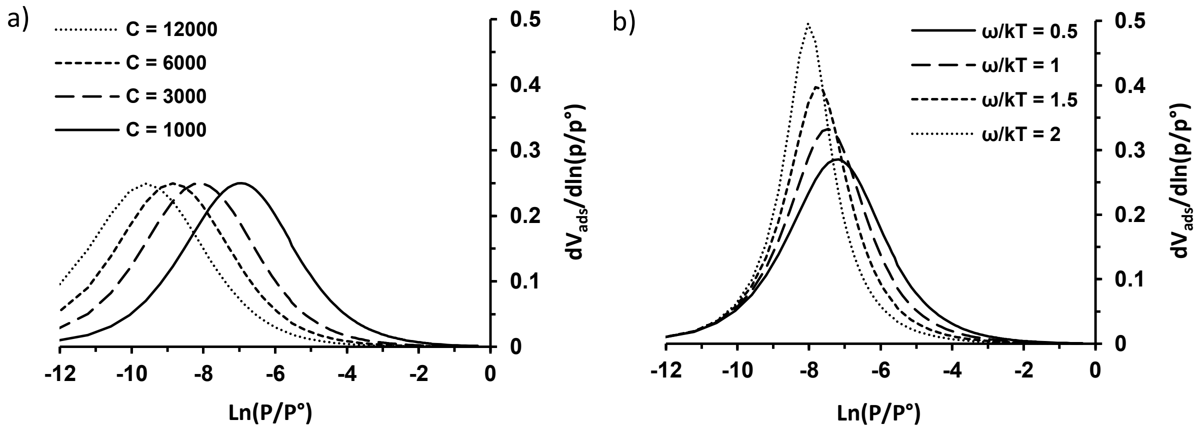

Figure S2. DIS modeling: the effect of parameters of BWT model on isotherm derivative shape: a) C – energy constant corresponding to the adsorbate-adsorbent interaction and b) ω/kT – parameter responsible for adsorbate-adsorbate interactions.

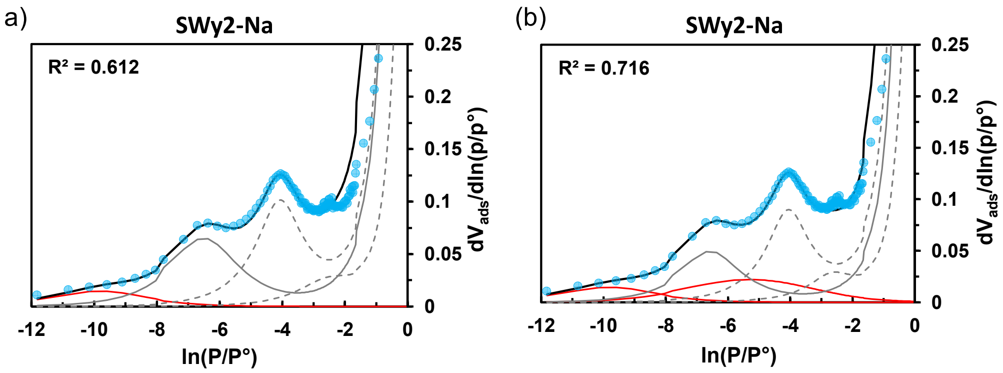

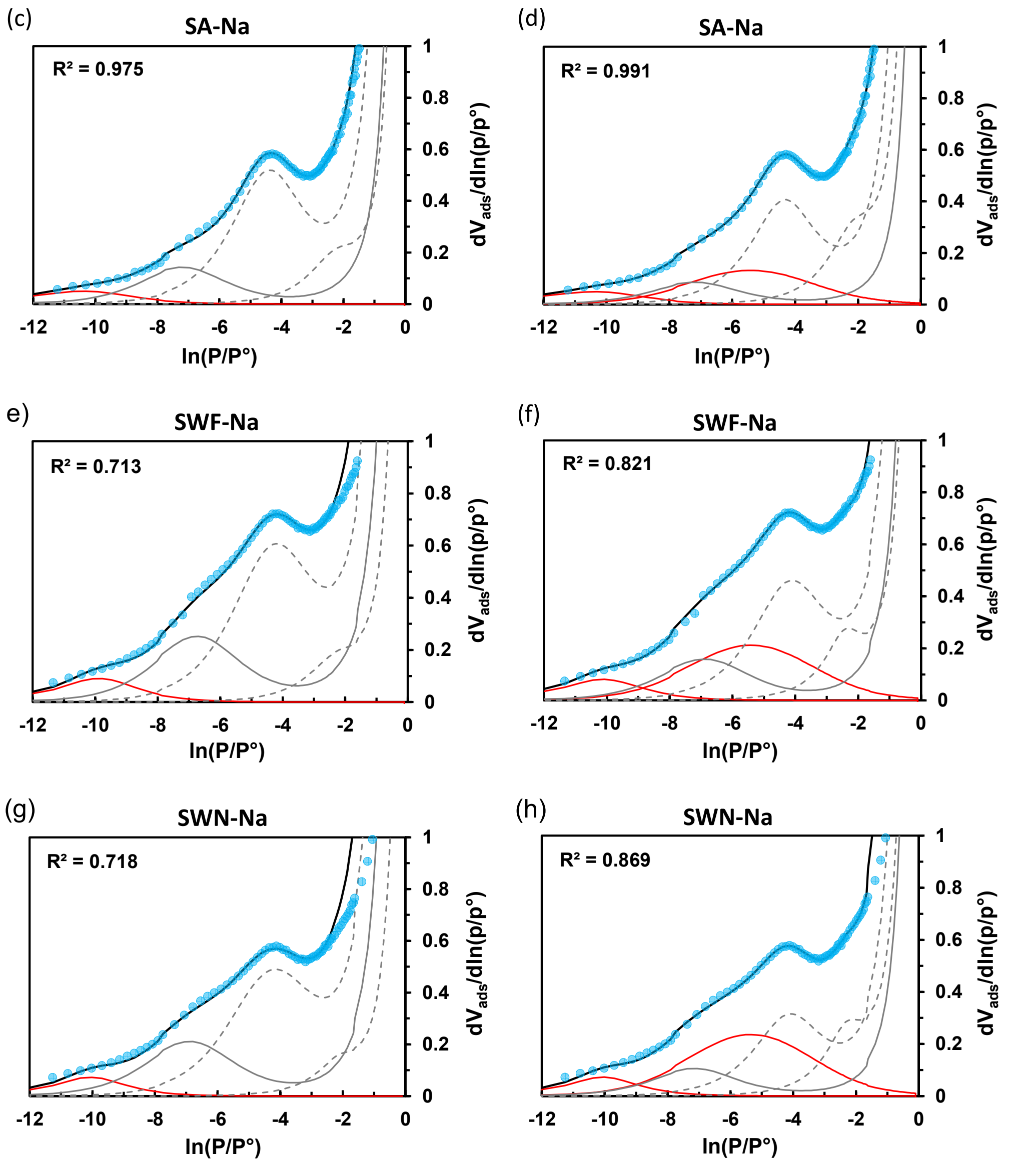

Figure S3. Derivatives of high-resolution Ar adsorption isotherms measured at 87 K (blue symbols) with their best-fit simulation with modified DIS method (black line) and individual components attributed to adsorption on the basal (grey dashed line) and lateral (grey continuous line) faces of clay particles and monolayer adsorption onto defectives sites at the edges and micropores (red line). Conventional (left) and modified (right) of DIS method.

Table S1. Comparison between results of conventional and modified DIS method applied to Ar adsorption isotherms at 87 K.

Component	Parameter	SWy2-Na	Modified DIS fit SWy2-Na	SA-Na	Modified DIS fit SA-Na	SWN-Na	Modified DIS fit SWN-Na	SWF-Na	Modified DIS fit SWF-Na
I	C	12000	12000	27000	27000	12000	12000	10500	15000
	ω/kT	0.3	0.3	0	0	1	1	2.2	0.7
II	C	350	320	1000	1000	800	900	2600	800
	ω/kT	1	1.28	0.3	0.3	0.2	0.4	0.1	0.2
III	C	25	300	55	320	58	320	260	320

	ω/kT	1.65	-1	0.75	-1	0.3	-1	0	-1
IV	C	8	23.2	5.6	45	4.4	38	42	43
	ω/kT	1	1.75	1.1	1	1.2	0.91	0.15	0.74
V	C	-	7	-	4.9	-	4.7	6.5	5
	ω/kT	-	1.4	-	1.13	-	1.38	0.94	1.5
% Edges		41.05	44.08	21.03	15.76	28.99	21.59	30.34	24.45
B/L		1.44	1.2684	3.75	5.34	2.45	3.63	2.2959	3.1

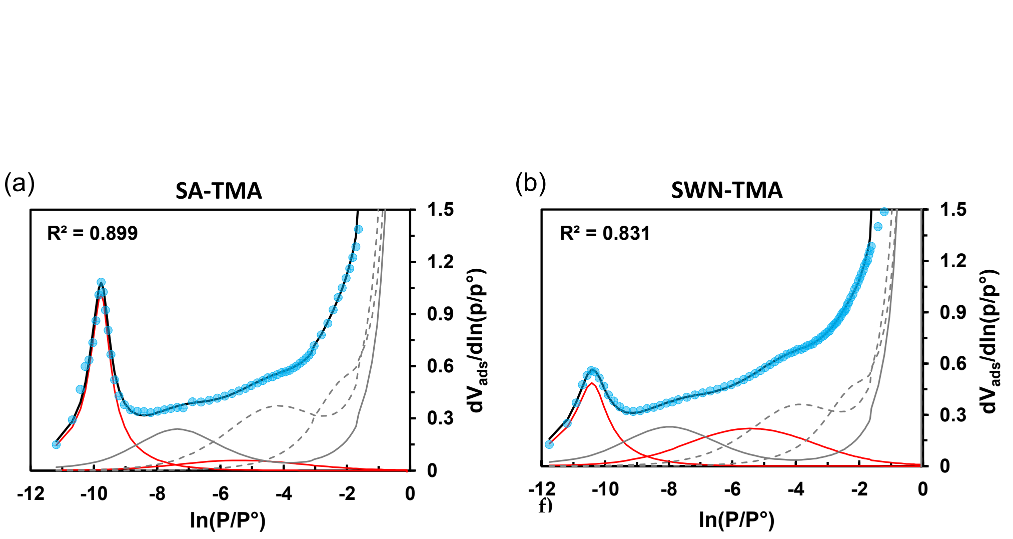

e)

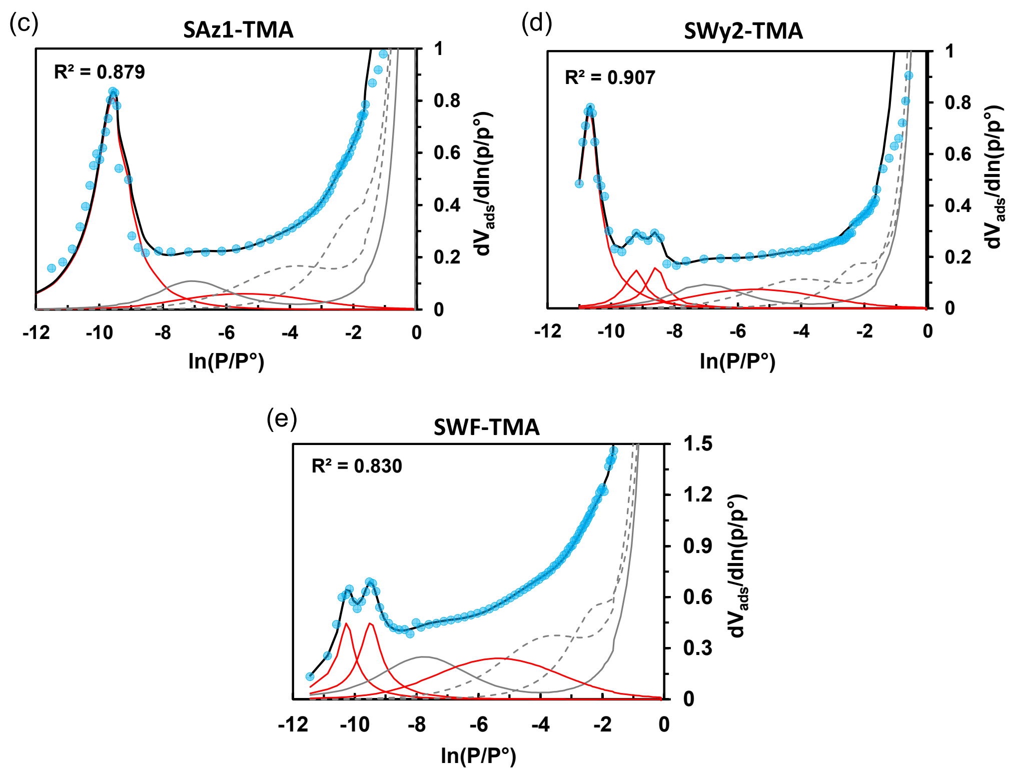

Figure S4. Derivatives of high-resolution Ar adsorption isotherms measured at 87 K for studied clays exchanged with TMA^+ (blue symbols) with their best-fit simulation with modified DIS method (black line) and individual components attributed to adsorption on the basal (grey dashed line) and lateral (grey continuous line) faces of clay particles and monolayer adsorption onto defectives sites at the edges (red line).

Table S2. Results of best-fit proposed of modified DIS method applied to Ar adsorption isotherms at 77 K for studied clays exchanged with TMA^+.

Component	Parameter	SAz1-TMA	SWy2-TMA	SA-TMA	SWN-TMA	SWF-TMA
I	C	4600	10700	4400	10500	6300
	ω/kT	2.2	2.72	2.7	2.2	2.9
II	C	600	2250	1200	2600	3300
	ω/kT	0.8	2.8	0.3	0	2.65
III	C	320	1150	320	320	1900

	ω/kT	-1	2.85	-1	-1	0.2
IV	C	50	580	70	50	320
	ω/kT	0	0.8	0	0.15	-1
V	C	5.4	320	5.5	6	44
	ω/kT	1	-1	1	1	0
VI	C	-	50	-	-	5.7
	ω/kT	-	0.22	-	-	1.16
VII	C	-	5	-	-	-
	ω/kT	-	1.3	-	-	-
% Edges		21.88	64.97	27.33	29.87	29
B/L		3.57	0.54	2.66	2.35	2.45

Table S3. Intra-particles microporosity surfaces for the exchanged clays deduced by the modified DIS method.

**Sample**		**$S_{\mu p}$ (m^2/g)**	**% $S_{\mu p}$**
SWy-2	Na+	4.7	15.7
	TMA+	115.7	44.2
SAz-1	Na+	11.1	9.5
	TMA+	124.9	43.4
SA	Na+	17.1	6.2
	TMA+	112.9	24.4
SWF	Na+	23.1	6.5
	TMA+	94.9	17.7
SWN	Na+	18.8	6.3
	TMA+	75.1	16.1
